# Prevalence and Predictors of Chemotherapy‐Related Cognitive Impairment in Elderly Lung Cancer Patients: A Cross‐Sectional Study

**DOI:** 10.1002/brb3.70594

**Published:** 2025-06-04

**Authors:** Wu XiuCen, Chen GuiHua, Li Qin, Tang Huan, Shen HuaPeng

**Affiliations:** ^1^ School of Nursing Chongqing Medical University Chongqing China; ^2^ Nursing Department The Second Affiliated Hospital of Chongqing Medical University Chongqing China; ^3^ Department of Respiratory Medicine The Second Affiliated Hospital of Chongqing Medical University Chongqing China

**Keywords:** chemotherapy‐related cognitive impairment, cognition, influencing factors, lung cancer, nursing

## Abstract

**Objective:**

To investigate the potential factors contributing to chemotherapy‐related cognitive impairment in elderly lung cancer patients and to offer insights for the creation of an intervention program aimed at enhancing the cognitive abilities of this vulnerable population.

**Methods:**

Three hundred thirty‐eight lung cancer patients from the departments of respiratory medicine, oncology, and thoracic surgery of two tertiary‐level hospitals in Chongqing from July 2023 to July 2024 were selected for the study via convenience sampling. A self‐designed General and Disease‐Related Information Questionnaire, Hospital Anxiety and Depression Scale (HADS), Brief Fatigue Inventory (BFI), Pittsburgh Sleep Quality Index (PSQI), Mini‐Mental State Examination (MMSE), and Functional Assessment of Cancer Therapy Cognitive Function (Fact‐Cog) were utilized for the questionnaire survey. Univariate analysis of variance and binary logistic regression were employed to analyze the factors influencing chemotherapy‐related cognitive impairment in elderly lung cancer patients.

**Results:**

Of the 338 participants, 300 (88.8%) were male and 38 (11.2%) were female, and 227 (67.2%) were between the ages of 60–69 years. The prevalence of cognitive impairment measured using the FACT‐Cog was 32.8%, and univariate analysis revealed that homeplace, drinking, the nature of occupation before retirement, tea‐drinking habits, leisure activities, ADL, pet ownership, HADS, BFI, PSQI, education level, hemoglobin value, and insurance were statistically significant (p < 0.05). Multifactorial analysis demonstrated that education level of junior high school (OR = 0.056, p < 0.05), education level of high school/secondary school (OR = 0.035, p < 0.05), education level of bachelor's degree/tertiary school (OR = 0.028, p < 0.05), and hemoglobin (OR = 0.981, p = 0.006) were protective factors for CRCI. Additionally, the nature of occupation before retirement (OR = 0.387, p = 0.005) and leisure activities (OR = 0.342, p = 0.001) were also found to be protective factors. Conversely, diagnosed anxiety‐depression (OR = 2.938, p = 0.003) and severe fatigue (OR = 3.465, p = 0.001) emerged as risk factors for CRCI.

**Conclusions:**

CRCI is the result of the intricate interplay of multiple potential factors and complex mechanisms. Healthcare professionals need to develop personalized intervention plans for patients based on their unique influencing factors, aiming to mitigate the adverse effects of risk factors on patients' cognitive functions. This approach can enhance patients' sense of self‐worth and satisfaction with life, ultimately improving the quality of life of elderly lung cancer patients in the long term.

## Graphical abstract

This cross‐sectional study assessed 338 elderly lung cancer patients to determine the prevalence and risk factors of chemotherapy‐related cognitive impairment (CRCI). The prevalence of CRCI was 32.8%. Protective factors included higher education levels, higher hemoglobin levels, pre‐retirement occupation, and leisure activities. Risk factors included diagnosed anxiety‐depression and severe fatigue. Findings highlight the need for personalized interventions to improve cognitive outcomes in this vulnerable population.

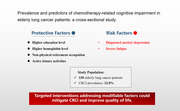

## 1 Introduction

Lung cancer remains the leading cause of cancer‐related deaths globally, with incidence and mortality rates ranking first in China in 2022 (Xia et al. 2022; Qin et al. 2022). Despite advancements in cancer diagnosis and treatment, lung cancer continues to significantly impact patients' quality of life and survival rates, and the 5‐year survival rate for lung cancer patients at stages N0‐N1 who receive surgical treatment ranges from 40.7% to 50%. Patients undergoing chemotherapy can achieve a 5‐year survival rate of 40–58% (Drevet et al. 2020; Nagasaka and Gadgeel 2018). These statistics indicate that many lung cancer patients now experience chronic survival with the disease (Howlader et al. 2020), and the influence of both the disease itself and treatment‐related side effects on patients' social functioning and quality of life has increasingly come to be recognized.

Chemotherapy, a common modality of cancer treatment, can have severe side effects on the central nervous system, including memory loss associated with hippocampal and frontal lobe dysfunction and impairment of attention, working memory, and strategic learning (Winocur et al. 2018). Chemotherapy‐related cognitive impairment (CRCI), also known as “chemobrain” or “chemofog,” is a well‐recognized clinical syndrome referring to cognitive changes occurring in non‐neurological cancer patients during or after chemotherapy (Lange et al. 2019; Silberfarb 1983). Some patients experience cognitive decline during or after chemotherapy, with reported rates ranging from 17% to 70% among tumor patients (Wefel et al. 2015). The mechanism behind CRCI may involve chemotherapeutic drugs causing damage to the central nervous system and neural precursor cells, which indirectly leads to changes in oxidative stress and inflammatory responses (Wefel et al. 2015; Li et al. 2019). Simo et al. conducted research exploring the changes in brain function among patients with different types of lung cancers after undergoing chemotherapy. Their findings indicate that chemotherapy alters brain functional connectivity in lung cancer patients, subsequently leading to cognitive decline.

CRCI negatively impacts various aspects of patients' quality of life, including autonomy, return to work, functional independence, social relationships, and treatment adherence (Schagen et al. 2022; Wefel et al. 2015). Paraskevi et al. (2021) conducted research on the quality of life and psychological stress of lung cancer patients using a questionnaire. Their findings revealed that patients with lung cancer experience higher levels of psychological stress and lower quality of life compared to the general population. Papadopoulos et al. (2019) demonstrated that a higher psychological burden in lung cancer patients undergoing chemotherapy is associated with sleep disturbances and increased fatigue. These studies highlight the interconnected presentation of somatic symptoms and psychological aspects of CRCI in lung cancer patients. Furthermore, they underscore the importance of screening for psychological factors to provide appropriate guidance in the presence of cognitive symptoms.

The influencing factors associated with CRCI in lung cancer patients are multifaceted. Studies (Luo et al. 2023) have shown that a high symptom load is a risk factor for CRCI in lung cancer patients, and systematic evaluations have indicated that advanced age is another influencing factor for CRCI in lung cancer patients (Ye et al. 2024). However, these studies have limitations. They did not account for lung cancer patients with pre‐existing cognitive deficits, and the cognitive assessment tools used were relatively homogeneous, focusing primarily on cancer‐related cognitive impairment (CRCI). Moreover, these studies did not include regular chemotherapy as an inclusion criterion for the study population, which may limit their applicability to the broader spectrum of lung cancer patients experiencing CRCI.

It's crucial to acknowledge that current research predominantly focuses on breast cancer patients, while studies on CRCI in lung cancer patients are still in their infancy (Iranzo et al. 2023; Koevoets et al. 2022). Most existing studies are cross‐sectional, and the assessment tools lack objectivity. The International Group on Cognition and Cancer recommends the use of standardized cognitive assessment tools, such as the FACT‐Cog or PROMIS cognitive ability and cognitive attention scale, to objectively measure CRCI (Deprez et al. 2018). Notably, subjective cognitive dysfunction may manifest before objective impairment, significantly affecting patients' daily self‐management and individual quality of life. In this study, researchers utilized the Mini‐Mental State Examination (MMSE) and the Functional Assessment of Cancer Therapy Cognitive Function (FACT‐Cog) to conduct early risk screening for CRCI in lung cancer patients. These assessments were administered twice to patients to ensure accurate determination of CRCI risk. Cognitive screening was employed to precisely identify instances of CRCI in patients, and a series of symptom burden factors related to CRCI in lung cancer patients were incorporated into the influencing factors. The primary goal was to explore interventions aimed at improving the quality of long‐term survival for patients through the examination of these influencing factors. This approach aims to enhance treatment compliance, boost the sense of self‐worth and life satisfaction of lung cancer patients, and provide a better understanding of the risk factors of CRCI in elderly lung cancer patients, ultimately contributing to the prevention and management of CRCI in elderly lung cancer patients.

## 2 Method

### 2.1 Study Design

**FIGURE 1:**
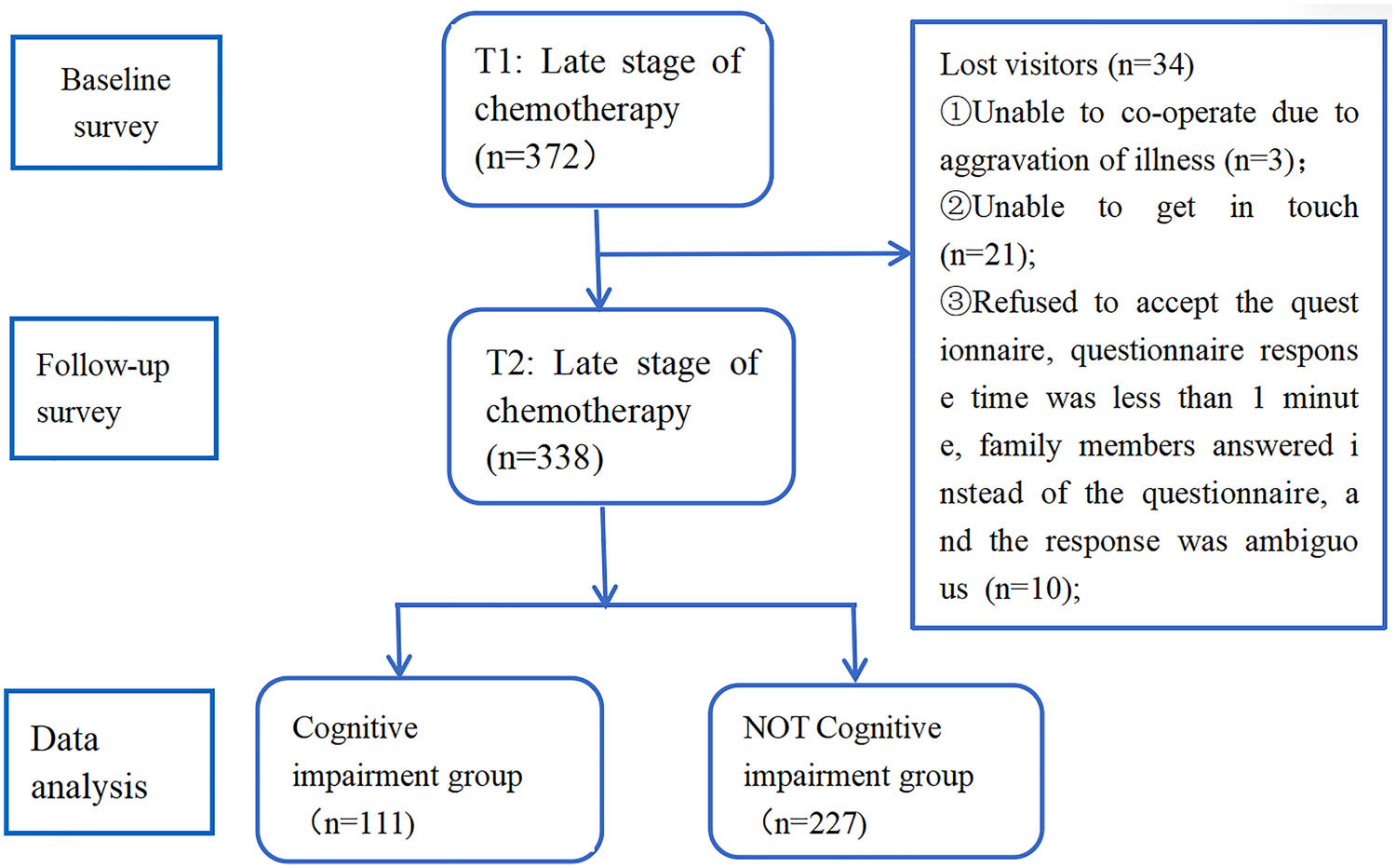
Data collection chart.

This study employed a cross‐sectional design utilizing convenience sampling. From July 2023 to July 2024, we conducted a survey among lung cancer patients in two hospitals, resulting in 338 completed questionnaires, yielding a recovery rate of 97.38%. Data entry was performed by two independent researchers using an Excel spreadsheet designed by the authors and validated by a third researcher. The process of participant selection and data collection is outlined in Figure 1.

### 2.2 Subjects of the Survey

The subjects of the survey consisted of 338 lung cancer patients selected from the departments of respiratory medicine, oncology, and thoracic surgery of two tertiary‐level A hospitals in Chongqing during the period from July 2023 to July 2024. The inclusion criteria were as follows: (1) meeting the lung cancer standards outlined in the Chinese Medical Association's Clinical Guidelines for Lung Cancer Diagnosis and Treatment (2023 edition) (Dong et al. 2023);(2) age ≥ 60 years; (3) receiving regular chemotherapy with at least 3 or more cycles; (4) possessing normal communication and understanding abilities, with an MMSE score of ≥ 25.

Exclusion criteria included (1) presence of brain metastases, (2) use of medications affecting cognitive function, (3) history of neurological diseases, psychiatric conditions, or psychotic episodes, and (4) pre‐existing cognitive disorders. Based on the Kendall sample estimation method, which recommends 5–10 times the number of variables for multivariate analysis, and considering a 20% non‐response rate and 20% invalid questionnaires, the estimated minimum sample size was N = 180, while the maximum was N = 360. The final sample size of 338 cases was determined. The study was approved by the Ethics Committee (20240809), and all respondents provided informed consent.

### 2.3 Survey Instruments

#### 2.3.1 General and Disease‐Related Information Questionnaire

The questionnaire was designed by the researcher and refined through group discussions, literature reviews, and expert opinions. It comprised 30 entries, including general information such as gender, age, marital status, medical insurance, education level, BMI, and homeplace, along with disease‐related information like tumor type, disease staging, and transfer.

#### 2.3.2 Hospital Anxiety and Depression Scale (HADS)

The Chinese version of the Hospital Anxiety and Depression Scale (HADS) was translated and revised by Xu et al. (1991). The scale comprises 14 items, with the odd‐numbered items assessing anxiety and the even‐numbered items assessing depression. Each item is rated on a 4‐point Likert scale ranging from 0 to 3. Scores of 0–7 indicate no symptoms, 8–10 suggest possible (borderline) anxiety or depression, and 11–21 indicate definite anxiety or depression. In a study of cancer patients, the total Cronbach’s alpha coefficient for the Chinese version of the HADS was 0.820 (Zhang et al. 2012).

#### 2.3.3 Brief Fatigue Inventory (BFI)

Developed by Mendoza et al. in 1999, the BFI assesses fatigue levels and their impact on daily functioning in cancer patients. It comprises nine items, with the first three evaluating fatigue over a 24‐hour period and the remaining six assessing its impact on life. Items were scored on a 0–10 scale, with 0 indicating “no fatigue or no effect” and 10 representing “most fatigue/complete effect imaginable.” Scores of 4–6 indicated moderate fatigue. The total score is the sum of individual item scores, with higher scores indicating more severe fatigue. The Cronbach's alpha coefficient for this scale in cancer patients was 0.944 (Gao et al. 2009).

#### 2.3.4 Pittsburgh Sleep Quality Index (PSQI)

Compiled by Buysse et al. in 1989, the PSQI evaluates patient sleep status over one month, covering seven items such as sleep efficiency, duration, and quality. The total score is 21 points, with 0–5 indicating very good sleep quality, 6–10 indicating fair sleep quality, 11–15 indicating poor sleep quality, and 16–21 indicating very poor sleep quality. Higher scores indicate poorer sleep quality. Liu et al. verified the scale's reliability, with sensitivity and specificity of 98.3% and 90.2%, respectively. The Cronbach's alpha coefficient for the overall scale was 0.84.

#### 2.3.5 Mini‐Mental State Examination (MMSE)

The Mini‐Mental State Examination (MMSE), developed by Folstein et al. (1975), is a widely used tool for screening cognitive impairment and dementia. It consists of 30 items assessing orientation, memory, attention and calculation, recall, and language abilities. Each correct response is scored 1 point, with a total score ranging from 0 to 30. A score below 24 suggests cognitive impairment. Diagnostic criteria are based on education level: 17–19 points for illiteracy, 20–23 points for primary school education, and 24–26 points for secondary school education or above. Scores below the lower limit are considered indicative of dementia, while those above the upper limit suggest normal cognitive function. The Cronbach's alpha coefficient for the Chinese version of the MMSE is 0.83 (Zhou et al. 2015).

#### 2.3.6 Functional Assessment of Cancer Therapy Cognitive Function (FACT‐Cog)

Developed by Wagner et al. in 2009 through qualitative interviews with cancer patients, FACT‐Cog assesses subjective cognitive functioning in cancer patients. It consists of four dimensions: perceived cognitive impairments (CogPCl), comments from others on cognitive function (CogOth), perceived cognitive abilities (CogPCA), and the impact of perceived cognitive impairment on quality of life (CoqQOL). The scale comprises 37 entries, each rated on a 5‐point scale. Higher total scores indicate better perceived cognitive functioning and quality of life. In this study, the CogPCI subscale was adopted, consisting of 20 items ranging from zero (“never”) to 4 (“how many times a day”). A total score below 54 indicates a self‐reported cognitive problem. Cronbach's alpha coefficients for the scale and subscales ranged from 0.87 to 0.96 (Li et al. 2015).

### 2.4 Data Collection and Quality Control Methods

In this study, lung cancer patients undergoing regular chemotherapy were prospectively followed up until the late stage of chemotherapy. Cognitive functions of the patients were assessed, and questionnaires were administered throughout the study. Prior to the commencement of the study, investigators underwent unified training to standardize the introduction and explanation of questions. Before completing the questionnaire, investigators explained to patients the purpose, content, confidentiality, anonymity, and requirements for filling it out. The investigators completed the baseline survey in the pre‐chemotherapy period of the patients, collecting basic information from the medical record system and screening patients based on regular chemotherapy criteria. Questionnaires were distributed to eligible lung cancer patients who provided informed consent. During the questionnaire completion process, investigators explained the meaning of entries to patients in real‐time. Patients retrieved the completed questionnaire upon finishing and had incorrectly filled‐out or omitted entries verified and supplemented. Questionnaire completion typically took 10–20 min. To distinguish normal cognitive aging, patients' cognition was screened in the pre‐chemotherapy period to exclude those with dementia. The second assessment of cognitive function was conducted after the chemotherapy cycle lasted for at least three cycles. Post‐chemotherapy assessments were performed using questionnaires, mailings, and telephone calls. Questionnaires with more than 10% missing data, not filled according to norms, or exhibiting illogicality were excluded. Missing values in questionnaires with less than 10% missing data were processed by calculating the average value of the entry.

### 2.5 Statistical Methods

Data entry and analysis were conducted using Excel 2010 for double‐entry verification and SPSS 25.0 software for statistical processing. Normality tests were performed using the Kolmogorov‐Smirnov (K‐S) test. Variables were determined to be normally distributed when the K‐S test yielded a p‐value greater than 0.05. Hemoglobin (HB) levels were found to follow a normal distribution. Normally distributed measurement data were expressed as mean ± standard deviation (x ± s), while categorical data were presented as frequency and percentage. Statistical analyses were conducted as follows: Independent samples t‐tests were used for comparing measurement data conforming to normal distributions between groups. Chi‐square tests were employed for comparing categorical data between groups. Rank data were analyzed using the Wilcoxon rank‐sum test. Binary logistic regression analysis was performed for multifactorial analyses. Statistical significance was determined at a two‐tailed p‐value < 0.05.

## 3 Results

### 3.1 General and Disease‐Related Information of the Investigated Subjects

According to the inclusion and exclusion criteria, a total of 338 lung cancer patients were included in this study. The age distribution of the patients ranged from 60–84 years old, with the largest number (122 cases, 36.1%) falling within the 65–69 years age stratum, followed by the 60–64 years group with 105 cases (31.1%). Only 6 patients (1.7%) were aged 80 or above. Gender distribution showed 38 (11.2%) females and 300 (88.8%) males. Notably, the probability of CRCI was higher in females (47.4%) compared to males (31.0%). Cognitive impairment status revealed that 111 cases (32.8%) exhibited chemotherapy‐related cognitive impairment (CRCI), while 227 patients (67.2%) had non‐chemotherapy‐related cognitive impairment. Approximately 30.5% of the patients had primary school education or less. Treatment modalities showed that most patients received combination chemotherapy (71.9%), and 180 (53.3%) were engaged in manual labor before retirement. Smoking behavior was reported by 192 (56.8%) patients, and alcohol consumption by 114 (33.7%). Prevalence rates of hypertension, diabetes mellitus, and coronary heart disease were 32.2%, 13.9%, and 7.1%, respectively. These demographic and disease‐related details are presented in Table 1.

**TABLE 1:** Comparative analysis of general and disease‐related information on CRCI in elderly lung cancer patients (n = 338).

Variables	Cognitive impairment (n = 227)	Not cognitive impairment(n = 111)	χ^2^/z/t Value	p Value
Age[n(%)]			−1.931	0.054
60‐64	78(34.4)	27(24.4)		
65‐69	80(35.2)	42(37.8)		
70‐74	50(22.0)	28(25.2)		
75‐79	14(6.2)	13(11.7)		
80‐84	5(2.2)	1(0.9)		
Gender[n(%)]			4.097	0.430
Male	207(91.2)	93(83.8)		
Female	20(8.8)	18(16.2)		
Homeplace[n(%)]			14.002	0.000
Urban areas	112(49.3)	31(27.9)		
Rural areas	115(50.7)	80(72.1)		
Education level [n(%)]			−6.932	0.000
Illiteracy	1(0.4)	9(8.2)		
Primary education	43(18.9)	50(45.1)		
Lower secondary education	88(38.8)	37(33.3)		
High school/secondary education	66(29.1)	12(10.8)		
Bachelor's degree/tertiary	29(12.8)	3(2.6)		
Marital status [n(%)]			1.800	0.180
Married	225(99.1)	4(3.6)		
Single or divorce	2(0.9)	107(96.4)		
Medical Insurance [n(%)]			17.792	0.000
Commercial insurance payment	163(71.8)	54(48.6)		
Social security payments	61(26.9)	56(50.5)		
Self‐paying	3(1.3)	1(0.9)		
Drinking [n(%)]			5.346	0.021
Yes	86(37.9)	28(25.2)		
No	141(62.1)	83(74.8)		
Smoking history [n(%)]			2.003	0.157
Yes	135(59.5)	57(51.4)		
No	92(40.5)	54(48.6)		
The nature of occupation before retirement [n(%)]			51.409	0.000
Brain work	137(60.4)	21(18.9)		
Non‐mental work	90(39.6)	90(81.1)		
Solitary [n(%)]			0.442	0.506
Yes	4(1.8)	4(3.6)		
No	223(98.2)	107(96.4)		
Tea drinking habit[n(%)]			4.831	0.028
Yes	141(62.1)	55(49.5)		
No	86(37.9)	56(50.5)		
Pet ownership[n(%)]			5.663	0.017
Yes	66(29.1)	19(17.1)		
No	161(70.9)	92(82.9)		
BMI [n(%)]			−0.893	0.372
Too light	14(6.2)	5(4.5)		
Normal	133(58.6)	74(66.7)		
Overweight	58(25.6)	24(21.6)		
Obese	22(9.7)	8(7.2)		
ADL [n(%)]			−4.486	0.000
Independence	19(8.4)	0(0.0)		
Mildly dependent	202(89.0)	98(88.3)		
Moderate dependence	3(1.3)	6(5.4)		
Heavy dependence	3(1.3)	7(6.3)		
Disease staging[n(%)]			−1.371	0.170
I Stage	14(6.2)	6(5.4)		
II stage	12(5.3)	5(4.5)		
III stage	59(26.0)	25(22.5)		
IV stage	114(50.2)	54(48.7)		
Non‐stageable tumors	28(12.3)	21(18.8)		
Tumor type[n(%)]			2.253	0.752
Squamous carcinoma	71(31.2)	36(32.5)		
Adenocarcinoma	110(48.5)	51(45.9)		
Small cell carcinoma	44(19.4)	22(19.8)		
Large cell carcinoma	2(0.9)	1(0.9)		
Undifferentiated carcinoma	0(0.0)	1(0.9)		
Hypertension[n(%)]			1.085	0.298
Yes	69(30.4)	40(36.0)		
No	158(69.6)	71(64.0)		
Diabetes[n(%)]			3.310	0.069
Yes	37(16.3)	10(9.0)		
No	190(83.7)	101(91.0)		
Coronary artery disease[n(%)]			0.720	0.396
Yes	18(7.9)	6(5.4)		
No	209(92.1)	105(94.6)		
Surgery[n(%)]			0.084	0.772
Yes	42(18.5)	22(19.8)		
No	185(81.5)	89(80.2)		
PaCO_2_[n(%)]			−0.247	0.805
Low	23(10.1)	14(12.6)		
Normal	182(80.2)	82(73.9)		
High	22(9.7)	15(13.5)		
Hb	117.81(20.616)	112.85(17.482)	2.180	0.030
HADS[n(%)]			−3.320	0.001
No	102(44.9)	30(27.1)		
Doubtful	71(31.3)	40(36.0)		
Yes	54(23.8)	41(36.9)		
BFI			−4.657	0.000
Mild	69(30.4)	21(19.0)		
Moderate	119(52.4)	40(36.0)		
Severe	39(17.2)	50(45.0)		
PSQI			−2.445	0.014
Well	43(19.0)	19(17.2)		
Not Bad	86(37.9)	25(22.5)		
General	60(26.4)	39(35.1)		
Poorly	38(16.7)	28(25.2)		
Combined with other therapies			2.235	0.135
Yes	169(74.4)	74(66.7)		
No	58(25.6)	37(33.3)		
Leisure activities			38.668	0.000
Yes	139(61.2)	28(25.2)		
No	88(38.8)	83(74.8)		
Transfer			0.005	0.942
Yes	140(61.7)	68(61.3)		
No	87(38.3)	43(38.7)		
Number of underlying diseases[n(%)]			−0.193	0.847
0	131(57.7)	63(56.8)		
1	70(30.8)	41(36.9)		
2	22(9.7)	4(3.6)		
≥3	4(1.8)	3(2.7)		

### 3.2 Levels of Chemotherapy‐Related Cognitive Impairment and Results of Univariate Analyses in Lung Cancer Patients

Patients were initially screened using MMSE, and a total of 338 lung cancer patients were subsequently screened for FACT‐cog cognitive impairment and included as subjects. In the CRCI group, univariate analysis revealed several factors influencing CRCI in lung cancer patients. Specifically, place of residence, history of alcohol consumption, occupation, ADL, tea‐drinking habits, leisure activities, pet ownership, levels of anxiety and depression, fatigue, sleep disorders, education level, hemoglobin value, and insurance status were identified as significant factors. A higher proportion of non‐brain workers before retirement in the CRCI group (81.1% vs. 18.9%, p < 0.001), Significantly higher rates of severe fatigue in the CRCI group (45.0% vs. 17.2%, p < 0.001), greater prevalence of diagnosed anxiety‐depression in the CRCI group (36.9% vs. 23.8%, p < 0.001). The proportion of patients with a high school education was higher in the non‐CRCI group than in the CRCI group (29.1% vs. 10.8%, p < 0.001).

### 3.3 Results of Binary Logistic Regression Analysis of Chemotherapy‐Related Cognitive Impairment in Lung Cancer Patients

CRCI was used as the dependent variable (assigned value: no cognitive impairment = 0, cognitive impairment = 1) with statistically significant variables from univariate analysis serving as independent variables. Binary logistic regression analysis revealed several factors influencing CRCI in lung cancer patients. These factors included pre‐retirement occupation, level of education, leisure activities, levels of anxiety and depression, fatigue, and hemoglobin (Hb) levels (P < 0.05). The values assigned to these independent variables are presented in Table 2, while the detailed results are shown in Table 3.

**TABLE 2:** Independent variable assignment methods.

Independent variable	Assignment method
	
Education level	Illiteracy = 0;Primary Education = 1;Lower Secondary Education = 2;High School/Secondary Education = 3;Bachelor's Degree/Tertiary = 4
ADL	independence = 0;mildly dependence= 1;Moderate dependence= 2; heavy dependence= 3
The nature of occupation before retirement	Non‐Mental Work = 0;Brain Work = 1
Tea drinking habit	No = 0;Yes = 1
Leisure activities	No = 0;Yes = 1
Homeplace	Rural Areas = 0;Urban Areas = 1
Drinking	No = 0;Yes = 1
Pet ownership	No = 0;Yes = 1
HADS	No = 0;Doubtful = 1;Yes = 2
Medical insurance	Medical Insurance = 0;Social Security Payments = 1;Self‐Paying = 2
BFI	Mild = 0;Moderate = 1;Severe = 2
PSQI	Well = 0;Not Bad = 1;General = 2;Poorly = 3

**TABLE 3:** Independent variable assignment.

Variables	Regression coefficient	Standard error	Wald χ^2^	P value	OR	95%CI
Constant	4.190	1.404	8.880	0.003		
Education level						
Lower secondary education	−2.891	1.124	6.605	0.010	0.056	0.003∼0.356
High school/secondary education	−3.345	1.181	8.009	0.005	0.035	0.002∼0.260
Bachelor's degree/tertiary	−3.589	1.308	7.507	0.006	0.028	0.001∼0.266
Hb	−0.019	0.007	7.076	0.008	0.981	0.967∼0.995
The nature of occupation before retirement	−0.949	0.367	6.708	0.010	0.387	0.186∼0.788
Leisure activities	−1.074	0.306	12.320	0.001	0.342	0.186∼0.619
HADS(take the absence of anxiety and depression as a reference)						
Yes	1.078	0.362	8.880	0.003	2.938	1.457∼6.048
BFI(use mild fatigue as a reference)						
Heavy fatigue	1.243	0.390	10.176	0.001	3.465	1.630∼7.566

## 4 Discussion

### 4.1 Lower Incidence of Chemotherapy‐Related Cognitive Impairment in Lung Cancer Patients With High Cognitive Reserve

Cognitive health is the development and maintenance of multidimensional cognitive structures that enable older adults to maintain social connectedness, a sustained sense of purpose, and the ability to function independently, recover function from illness or injury, and cope with residual functional deficits (Clare et al. 2017). Chemotherapy‐related cognitive impairment (CRCI) has a significant negative impact on patients' daily life, occupational functioning, and social interactions. It also affects treatment adherence and rehabilitation outcomes (Bifulco et al. 2012; Binarelli et al. 2023). Cognitive impairment is common among lung cancer patients. According to Takemura et al. (2022), 35.4% of 226 patients reported attention deficits and 58.4% had memory impairments after cancer treatment. To mitigate the effects of controllable factors on patient cognition, it is essential to adopt a comprehensive approach tailored to the individual needs of each patient.

The present study found that patients with a high level of education and those whose pre‐retirement occupations were cerebral in nature had a lower risk of CRCI, which aligns with Clare et al.’s findings. Cognitive reserve, indexed by education and occupational complexity, serves as an important mediator of the association between lifestyle factors and cognition. This suggests that occupation and education influence patients' cognitive functioning and lifestyles through cognitive reserve. Patients whose pre‐retirement occupations were cerebral in nature and highly educated lung cancer patients tended to have higher individual cognitive reserve and were able to cope with cognitive challenges through early cognitive stimulation and intensive adaptive strategies. Studies have demonstrated (Zhou et al. 2024) that highly educated individuals exhibit greater psychological adaptability and superior information acquisition and processing skills. The current study revealed that highly educated patients had a lower probability of cognitive decline yet perceived more significant cognitive decline than less educated CRCI patients. This finding corroborates the research conducted by Ophey et al., who attributed this phenomenon to several factors. Firstly, individuals with higher levels of education tend to have higher standards of self‐perception and self‐expectation. As a result, they are more sensitive and rigorous in monitoring and evaluating their cognitive functions. When cognitive decline occurs, they can detect these changes earlier and more accurately and may perceive these changes more strongly. Secondly, people with rich educational backgrounds are typically engaged in jobs or daily activities that require higher cognitive abilities, such as complex analyses, decision‐making, and learning new knowledge. Once cognitive decline occurs, there is a greater likelihood that it will affect daily performance and quality of life, resulting in a more pronounced perceived decline. Furthermore, the high value placed on cognitive health by highly educated individuals led to greater anticipation and anxiety of cognitive decline. Consequently, healthcare professionals should not only focus on the cognitive situation of the low‐educated lung cancer group but also address the more psychologically altered, highly educated group.

In this study, we found no association between age and CRCI in lung cancer patients, which contrasts with the findings reported by Ye et al. Several factors may contribute to this discrepancy. First, CRCI is influenced by life experience and social environment, which can vary significantly across different generations. Differences in average education levels and mental outlook among individuals born in different eras may also play a role. Additionally, the elderly may exhibit greater mental resilience due to their exposure to various health challenges over time. These factors suggest that the relationship between age and CRCI cannot be simplified or directly attributed to age itself. Rather, it appears that age is just one of many complex influences on CRCI in lung cancer patients. Further research is needed to fully understand the nuanced interactions between age, life experience, and cognitive function in the context of lung cancer.

### 4.2 CRCI in Lung Cancer Patients is Affected by a Variety of Psychosocial Factors, and Symptom Awareness Needs to be Raised Among Patients

Most of the six key domains of cognitive functioning as defined by the Diagnostic and Statistical Manual of Mental Disorders, Fifth Edition (DSM‐5), are covered (Sachdev et al. 2014), with the social‐cognitive domain—encompassing emotion recognition and theory of mind—playing an important role in the overall psychosocial well‐being of the patient and influencing the intertwined presentation of somatic disorders and emotional symptoms. The present study suggests that fatigue and anxiety‐depression are influential factors in CRCI, which act synergistically and present as symptom clusters. The cognitive debt hypothesis (suggesting that repeated negative thinking interacts with the APOE‐e4 gene, which is closely associated with Alzheimer's disease, thereby increasing the risk of cognitive impairment) (Karim et al. 2021) suggests that prolonged preoccupation with negative events exacerbates the impediments to self‐psychological regulation and makes it difficult for patients to cope with the challenges posed by cancer. Consistent with the results of existing studies on CRCI associated with patients' symptom burden, especially fatigue and anxiety‐depression levels (Eggen et al. 2022; Ehrenstein et al. 2023). The reasons considered are the systemic toxicity of chemotherapeutic agents as well as direct neurotoxic effects, chronic inflammation as a relevant mechanism for (CRCI), and the biological basis for the development of symptom clusters (Li et al. 2022). In addition, economic toxicity is a challenge to patients' psychological tolerance, and economic toxicity increases the source of anxiety in patients. Identifying the factors that predict CRCI symptom clusters in lung cancer patients is of great clinical significance for accurate care and improving patients' quality of life, and early psychological screening of lung cancer patients is extremely important. Healthcare professionals should pay close attention to patients' negative emotions, reduce their symptom burden, alleviate their anxiety and depression, and improve their perception of healthy behaviors.

The present study found an association between hemoglobin and CRCI, which is consistent with the results of a meta‐analysis of anemia and the risk of cognitive impairment, where anemia accelerates cognitive decline and contributes to an increased risk of developing cognitive impairment (Kung et al. 2021). This association is believed to occur due to the reduction of cerebral perfusion in the presence of prolonged cerebral hypoxia and the potential for structural damage to brain tissues when oxygen supply is insufficient to meet brain metabolic demands, ultimately leading to brain functional degradation and cognitive dysfunction.

Early intervention therapy can remodel the cerebral cortex and brain tissues damaged by chemotherapy, potentially reducing or restoring impaired cognitive function (Tapia et al. 2023). Cognitive‐behavioral therapy has been shown to improve patients' anxiety and depression, increase treatment adherence, and enhance overall quality of life. Virtual reality‐based home‐based interventions offer a convenient, easily accessible treatment option, allowing patients to arrange their own training time and place. These interventions have produced positive changes in terms of patient anxiety/depression, fatigue, and stress symptoms (Bray et al. 2017). Healthcare professionals should promote better disengagement from the patient role, maintaining participation in valued activities and enhancing self‐esteem. They should also facilitate integration into the community and the workplace for lung cancer patients, subject to individual conditions. Patients should strive to improve communication and awareness of healthy behaviors, adopt beneficial lifestyles, and engage in recreational activities when their condition permits.

### 4.3 Promote Healthy Behaviors, Improve Self‐Management and Reduce Social Alienation in Lung Cancer Patients

The ASCO guidelines (Ligibel et al. 2022) suggest that exercise during cancer treatment leads to improvements in cardiorespiratory fitness, strength, fatigue, and other patient‐reported outcomes. However, the present study found that motivation and enthusiasm for exercise were generally low in lung cancer patients. Furthermore, leisure activities in elderly lung cancer patients did not typically take the form of regular physical activity but rather recreational programs (such as board activities, photography, and painting) and social activities (like dancing and traveling). This finding aligns with the conceptual explanatory model constructed by Cuevas et al. using the ProAdapt framework, which comprises four interconnected themes: symptoms, healthcare, self‐perception, and relationships. This framework integrates patients' diverse experiences to present a comprehensive view of the complex process of adapting to subjective cognitive dysfunction while highlighting the role of relationships in patients' attitudes toward coping with cognitive problems and improvements in cognitive functioning. Studies have demonstrated (Koevoets et al. 2022; Wefel et al. 2015; Xu et al., 2019) that both physical exercise and cognitive training increase neuroplasticity, with regular brain activity serving as a neuroprotective measure by boosting cognitive reserve. Relationships, social participation, and daily functioning are associated with better quality of life. Systematic evaluations of the social needs of older adults have identified the necessity for reciprocal relationships, i.e., the need to connect and belong to a network of social relationships (Huizenga et al. 2022). Patients need to be empowered with more knowledge and power to enable them to be more proactive in managing their own health. A strong social support network can assist patients in better coping with anxiety and uncertainty, thereby enhancing their ability to manage their illness (Gomersall et al. 2015). This suggests that the lung cancer CRCI community should receive clearer information about decision‐sharing processes, enabling patients to participate in shared decision‐making and reduce social alienation, thus improving self‐perception. Cognitive training can be achieved by elderly lung cancer patients through participation in activities such as chess and ball games, as well as listening to news messages daily, when their condition permits. These measures provide specific strategies and support for developing a sense of independence and empowerment.

## 5 Limitation

There are several limitations of this study. First, the samples were only from two hospital districts of a tertiary care hospital, which is a small sample size and limited geographical representation, which may affect the generalizability of the study results among elderly lung cancer patients in different regions. In addition, the study did not consider the potential impact of different chemotherapy regimens on cognitive outcomes. Detailed recording and analysis of the changes in cognitive function in each group of patients will help to more comprehensively understand the cognitive influencing factors in the course of chemotherapy in elderly patients with lung cancer and provide a more accurate reference basis for clinical treatment.

## 6 Conclusion

Utilizing both subjective and objective measures, this study revealed that CRCI is a multifaceted phenomenon characterized by mediating factors related to cancer and treatment, as well as moderating factors linked to lifestyle and health. The investigation identified several influential factors contributing to CRCI in lung cancer patients, including pre‐retirement occupation, educational level, hemoglobin levels, leisure activities, anxiety and depression levels, and fatigue. These findings demonstrate an intricate interplay of somatic and emotional symptoms in CRCI. The identification of these influencing factors provides valuable insights for early intervention in identifying risk factors and developing preventive strategies, potentially mitigating the cognitive impacts of chemotherapy.

However, this study gave limited attention to social determinants such as socioeconomic environment and cultural factors. It is recommended that future studies employ standardized research designs, cognitive testing protocols, and analytical methods to elucidate the mediating and moderating pathways of these influencing factors. This approach would lead to a more comprehensive understanding of the various factors at play. Interdisciplinary collaboration is crucial to deepen our comprehension of the essential characteristics of CRCI and to elucidate the relationships and pathways between different influencing factors. Additionally, more interventional studies are necessary to reduce the adverse effects of risk factors on patients' cognitive functioning. Such efforts could contribute to enhancing patients' sense of self‐worth and life satisfaction by addressing the complex interplay of factors influencing CRCI in lung cancer patients.

## Author Contributions

**Wu XiuCen**: writing – review and editing; methodology; investigation; conceptualization. **Chen GuiHua**: formal analysis; supervision; project administration. **Li Qin**: writing – review and editing; writing – original draft. **Tang Huan**: writing – review and editing; methodology; conceptualization. **Shen HuaPeng**: writing – review and editing; project administration; methodology; conceptualization.

## Ethics Statement

The authors have nothing to report.

## Conflicts of Interest

The authors declare no conflicts of interest.

### Peer Review

The peer review history for this article is available at https://publons.com/publon/10.1002/brb3.70594.

## Data Availability

Data included in article/supp. material/referenced in the article.

## References

[brb370594-bib-0001] Bifulco, G. , N. De Rosa , M. L. Tornesello , et al. 2012. “Quality of Life, Lifestyle Behavior and Employment Experience: A Comparison Between Young and Midlife Survivors of Gynecology Early Stage Cancers.” Gynecologic Oncology 124, no. 3: 444–451. 10.1016/j.ygyno.2011.11.033. 22119994

[brb370594-bib-0002] Binarelli, G. , M. Duivon , F. Joly , D. Ahmed‐Lecheheb , and M. Lange . 2023. “Cancer‐Related Cognitive Impairment: Current Perspectives on the Management of Cognitive Changes Following Cancer Treatment.” Expert Review of Neurotherapeutics 23, no. 3: 249–268. 10.1080/14737175.2023.2187288. 36951414

[brb370594-bib-0003] Bray, V. J. , H. M. Dhillon , M. L. Bell , et al. 2017. “Evaluation of a Web‐Based Cognitive Rehabilitation Program in Cancer Survivors Reporting Cognitive Symptoms after Chemotherapy.” Journal of Clinical Oncology 35, no. 2: 217–225. 10.1200/JCO.2016.67.8201. 28056205

[brb370594-bib-0004] Buysse, D. J. , C. R. Reynolds , T. H. Monk , S. R. Berman , and D. J. Kupfer . 1989. “The Pittsburgh Sleep Quality Index: A New Instrument for Psychiatric Practice and Research.” Psychiatry Research 28, no. 2: 193–213. 10.1016/0165-1781(89)90047-4. 2748771

[brb370594-bib-0005] Clare, L. , Y. T. Wu , J. C. Teale , et al. 2017. “Potentially Modifiable Lifestyle Factors, Cognitive Reserve, and Cognitive Function in Later Life: A Cross‐Sectional Study.” Plos Medicine 14, no. 3: e1002259. 10.1371/journal.pmed.1002259. 28323829 PMC5360216

[brb370594-bib-0006] Cuevas, H. , E. Heitkemper , and J. Kim . 2024. “Subjective Cognitive Dysfunction in Chronic Illness: A Systematic Review and Meta‐Synthesis.” Western Journal of Nursing Research 46, no. 9: 708–724. 10.1177/01939459241272039. 39158016 PMC11380369

[brb370594-bib-0007] Deprez, S. , S. R. Kesler , A. J. Saykin , D. Silverman , M. B. de Ruiter , and B. C. McDonald . 2018. “International Cognition and Cancer Task Force Recommendations for Neuroimaging Methods in the Study of Cognitive Impairment in Non‐CNS Cancer Patients.” JNCI‐Journal of the National Cancer Institute 110, no. 3: 223–231. 10.1093/jnci/djx285. PMC6658857 29365201

[brb370594-bib-0008] Dong, D. , Y. H. Huang , Y. J. Zhang , and H. C. Li . 2023. “Interpretation of the Chinese Medical Association Lung Cancer Clinical Guidelines (2023 Edition).” Chinese Clinical Journal of Thoracic and Cardiovascular Surgery 30, no. 11: 1533–1538. 10.1007/s00520-024-08873-w.

[brb370594-bib-0009] Drevet, G. , M. Duruisseaux , J. M. Maury , et al. 2020. “Lung Cancer Surgical Treatment After Solid Organ Transplantation: A Single Center 30‐Year Experience.” Lung Cancer 139: 55–59. 10.1016/j.lungcan.2019.10.023. 31739183

[brb370594-bib-0010] Eggen, A. C. , N. M. Richard , I. Bosma , et al. 2022. “Factors Associated With Cognitive Impairment and Cognitive Concerns in Patients With Metastatic Non‐Small Cell Lung Cancer.” Neuro‐Oncology Practice 9, no. 1: 50–58. 10.1093/nop/npab056. 35087675 PMC8789294

[brb370594-bib-0011] Ehrenstein, J. K. , S. van Zon , S. Duijts , et al. 2023. “Trajectories of Cognitive Symptoms and Associated Factors in Cancer Survivors After Return to Work: An 18‐Month Longitudinal Cohort Study.” Journal of Cancer Survivorship 17, no. 2: 290–299. 10.1007/s11764-022-01190-3. 35312951 PMC10036271

[brb370594-bib-0050] Folstein, M. F. , S. E. Folstein , and P. R. McHugh . 1975. “Mini‐mental state: A practical method for grading the cognitive state of patients for the clinician [Journal Article].” Journal of Psychiatric Research, 12, no. 3: 189–198. 10.1016/0022-3956(75)90026-6. 1202204

[brb370594-bib-0012] Gao, L. P. , X. Q. Zhu , H. Zhao , H. M. Jiao , and D. X. Chen . 2009. “Studies on Internal Consistency and Test‐Retest Reliability of Brief Fatigue Inventory in Cancer Patients.” PLA Nursing Journal 26, no. 8: 1–3. 10.3969/j.issn.1008-9993.2009.08.001.

[brb370594-bib-0013] Gomersall, T. , A. Astell , L. Nygård , A. Sixsmith , A. Mihailidis , and A. Hwang . 2015. “Living with Ambiguity: A Metasynthesis of Qualitative Research on Mild Cognitive Impairment.” The Gerontologist 55, no. 5: 892–912. 10.1093/geront/gnv067. 26315317 PMC4580312

[brb370594-bib-0014] Howlader, N. , G. Forjaz , M. J. Mooradian , et al. 2020. “The Effect of Advances in Lung‐Cancer Treatment on Population Mortality.” New England Journal of Medicine 383, no. 7: 640–649. 10.1056/NEJMoa1916623. 32786189 PMC8577315

[brb370594-bib-0015] Huizenga, J. , A. Scheffelaar , A. Fruijtier , J. P. Wilken , N. Bleijenberg , and T. Van Regenmortel . 2022. “Everyday Experiences of People Living With Mild Cognitive Impairment or Dementia: A Scoping Review.” International Journal of Environmental Research and Public Health 19, no. 17: 10828. 10.3390/ijerph191710828. 36078544 PMC9518176

[brb370594-bib-0016] Iranzo, P. , A. Callejo , J. Arbej , S. Menao , D. Isla , and R. Andrés . 2023. “Risk Factors for Cancer‐Related Cognitive Impairment in Breast and Colorectal Cancer Patients Who Undergo Chemotherapy.” Anales Del Sistema Sanitario De Navarra 46, no. 2:e1040. 10.23938/ASSN.1040. 37594060 PMC10498134

[brb370594-bib-0017] Karim, H. T. , M. Ly , G. Yu , et al. 2021. “Aging Faster: Worry and Rumination in Late Life Are Associated With Greater Brain Age.” Neurobiology of Aging 101: 13–21. 10.1016/j.neurobiolaging.2021.01.009. 33561786 PMC8122027

[brb370594-bib-0018] Koevoets, E. W. , S. B. Schagen , M. B. de Ruiter , et al. 2022. “Effect of Physical Exercise on Cognitive Function After Chemotherapy in Patients With Breast Cancer: A Randomized Controlled Trial (PAM study).” Breast Cancer Research 24, no. 1: 36. 10.1186/s13058-022-01530-2. 35619188 PMC9135390

[brb370594-bib-0019] Kung, W. M. , S. P. Yuan , M. S. Lin , et al. 2021. “Anemia and the Risk of Cognitive Impairment: An Updated Systematic Review and Meta‐Analysis.” Brain Sciences 11, no. 6. 10.3390/brainsci11060777. PMC8231247 34208355

[brb370594-bib-0020] Lange, M. , F. Joly , J. Vardy , et al. 2019. “Cancer‐Related Cognitive Impairment: An Update on state of the Art, Detection, and Management Strategies in Cancer Survivors.” Annals of Oncology 30, no. 12: 1925–1940. 10.1093/annonc/mdz410. 31617564 PMC8109411

[brb370594-bib-0021] Li, J. , W. Gao , L. M. Sun , A. J. Wang , Y. L. Bu , and F. L. Cao . 2015. “Reliability and Validity of the Chinese Version of the Functional Assessment of Cancer Therapy‐Cognitive Function in Chinese Women With Breast Cancer.” Chinese Journal of Practical Nursing 31, no. 33: 2554–2556. 10.3760/cma.j.issn.1672-7088.2015.33.017.

[brb370594-bib-0023] Li, S. , and C. S. Xie . 2019. “Progress of Research on the Pathogenesis and Treatment of Chemoencephalography.” Journal of Oncology 25, no. 04: 355–358. 10.11735/j.issn.1671-170X.2019.04.B014.

[brb370594-bib-0022] Li, N. , J. Lu , D. Xia , et al. 2022. “Serum Biomarkers Predict Adjuvant Chemotherapy‐associated Symptom Clusters in Radical Resected Colorectal Cancer Patients.” Journal of Gastrointestinal Oncology 13, no. 1: 197–209. 10.21037/jgo-21-904. 35284113 PMC8899734

[brb370594-bib-0024] Ligibel, J. A. , K. Bohlke , A. M. May , et al. 2022. “Exercise, Diet, and Weight Management During Cancer Treatment: ASCO Guideline.” Journal of Clinical Oncology 40, no. 22: 2491–2507. 10.1200/JCO.22.00687. 35576506

[brb370594-bib-0025] Liu, X. C. , M. Q. Tang , L. Hu , et al. 1996. “Reliability and Validity of the Pittsburgh Sleep Quality Index.” Chinese Journal of Psychiatry 29, no. 02: 103–107. https://link.cnki.net/urlid/51.1492.R.20230928.1638.024.

[brb370594-bib-0026] Luo, J. , R. Liu , Y. Luo , et al. 2023. “The High Burden of Symptoms Associated With Cognitive Impairment in Lung Cancer Patients: A Latent Class Analysis.” Asia‐Pacific Journal of Oncology Nursing 10, no. 4: 100200. 10.1016/j.apjon.2023.100200. 36890861 PMC9988398

[brb370594-bib-0027] Mendoza, T. R. , X. S. Wang , C. S. Cleeland , et al. 1999. “The Rapid Assessment of Fatigue Severity in Cancer Patients: Use of the Brief Fatigue Inventory.” Cancer 85, no. 5: 1186–1196. 10.1002/(sici)1097-0142(19990301)85:5<1186::aid-cncr24>3.0.co;2-n. 10091805

[brb370594-bib-0028] Nagasaka, M. , and S. M. Gadgeel . 2018. “Role of Chemotherapy and Targeted Therapy in Early‐Stage Non‐Small Cell Lung Cancer.” Expert Review of Anticancer Therapy 18, no. 1: 63–70. 10.1080/14737140.2018.1409624. 29168933 PMC6863145

[brb370594-bib-0029] Ophey, A. , K. Wirtz , S. Wolfsgruber , et al. 2024. “Mid‐ and Late‐Life Lifestyle Activities as Main Drivers of General and Domain‐Specific Cognitive Reserve in Individuals With Parkinson's Disease: Cross‐Sectional and Longitudinal Evidence From the LANDSCAPE Study.” Journal of Neurology 271, no. 8: 5411–5424. 10.1007/s00415-024-12484-0. 38951175 PMC11319368

[brb370594-bib-0030] Papadopoulos, D. , M. Kiagia , A. Charpidou , I. Gkiozos , and K. Syrigos . 2019. “Psychological Correlates of Sleep Quality in Lung Cancer Patients Under Chemotherapy: A Single‐Center Cross‐Sectional Study.” Psycho‐Oncology 28, no. 9: 1879–1886. 10.1002/pon.5167. 31264308

[brb370594-bib-0031] Prapa, P. , I. V. Papathanasiou , V. Bakalis , F. Malli , D. Papagiannis , and E. C. Fradelos . 2021. “Quality of Life and Psychological Distress of Lung Cancer Patients Undergoing Chemotherapy.” World Journal of Oncology 12, no. 2‐3: 61–66. 10.14740/wjon1371. 34046100 PMC8139742

[brb370594-bib-0032] Qin, N. , H. X. Ma , and G. F. Qin . 2022. “Annual Progress in Epidemiological Studies of Lung Cancer 2022.” Chinese Medical Journal 103, no. 14: 1068–1073. 10.3760/cma.j.cn112137-20221213-02640.

[brb370594-bib-0033] Sachdev, P. S. , D. Blacker , D. G. Blazer , et al. 2014. “Classifying Neurocognitive Disorders: The DSM‐5 Approach.” Nature Reviews Neurology 10, no. 11: 634–642. 10.1038/nrneurol.2014.181. 25266297

[brb370594-bib-0034] Schagen, S. B. , A. S. Tsvetkov , A. Compter , and J. S. Wefel . 2022. “Cognitive Adverse Effects of Chemotherapy and Immunotherapy: Are Interventions Within Reach?” Nature Reviews Neurology 18, no. 3: 173–185. 10.1038/s41582-021-00617-2. 35140379

[brb370594-bib-0035] Silberfarb, P. M. 1983. “Chemotherapy and Cognitive Defects in Cancer Patients.” Annual Review of Medicine 34: 35–46. 10.1146/annurev.me.34.020183.000343. 6344764

[brb370594-bib-0036] Simó, M. , X. Rifà‐Ros , L. Vaquero , et al. 2018. “Brain Functional Connectivity in Lung Cancer Population: An Exploratory Study.” Brain Imaging and Behavior 12, no. 2: 369–382. 10.1007/s11682-017-9697-8. 28290076

[brb370594-bib-0037] Takemura, N. , M. H. Ho , D. Cheung , and C. C. Lin . 2022. “Factors Associated With Perceived Cognitive Impairment in Patients With Advanced Lung Cancer: A Cross‐Sectional Analysis.” Supportive Care in Cancer 30, no. 11: 9607–9614. 10.1007/s00520-022-07377-9. 36178636

[brb370594-bib-0038] Tapia, J. L. , M. T. Taberner‐Bonastre , D. Collado‐Martínez , A. Pouptsis , M. Núñez‐Abad , and J. A. Duñabeitia . 2023. “Effectiveness of a Computerized Home‐Based Cognitive Stimulation Program for Treating Cancer‐Related Cognitive Impairment.” International Journal of Environmental Research and Public Health 20, no. 6. 10.3390/ijerph20064953. PMC10049401 36981862

[brb370594-bib-0039] Wagner, L. , J. J. Sweet , Z. Butt , J. Lai , and D. Cella . 2009. “Measuring Patient Self‐Reported Cognitive Function: Qualitative Findings and Initial Development of the Functional Assessment of Cancer Therapy‐Cognitive Function (FACT‐COG) Instrument.” Journal of Supportive Oncology 7, no. 6: W32–W39.

[brb370594-bib-0040] Wefel, J. S. , S. R. Kesler , K. R. Noll , and S. B. Schagen . 2015. “Clinical Characteristics, Pathophysiology, and Management of Noncentral Nervous System Cancer‐Related Cognitive Impairment in Adults.” CA‐A Cancer Journal for Clinicians 65, no. 2: 123–138. 10.3322/caac.21258. 25483452 PMC4355212

[brb370594-bib-0042] Winocur, G. , I. Johnston , and H. Castel . 2018. “Chemotherapy and Cognition: International Cognition and Cancer Task Force Recommendations for Harmonising Preclinical Research.” Cancer Treatment Reviews 69: 72–83. 10.1016/j.ctrv.2018.05.017. 29909223

[brb370594-bib-0043] Xia, C. , X. Dong , H. Li , et al. 2022. “Cancer Statistics in China and United States, 2022: Profiles, Trends, and Determinants.” Chinese Medical Journal 135, no. 5: 584–590. 10.1097/CM9.0000000000002108. 35143424 PMC8920425

[brb370594-bib-0044] Xu, J. M. 1991. “Somatisation of Anxiety and Depression.” Shanghai Psychiatry 3, no. 1: 6–7.

[brb370594-bib-0045] Xu, Z. Y. , and Q. H. Song . 2019. “Effects of Long‐Term Group Intellectual Sports Activities on Cognitive Function and Daily Mood in Older Adults.” Chinese Journal of Gerontology 39, no. 15: 3693–3696. 10.3969/j.issn.1005-9202.2019.15.030.

[brb370594-bib-0046] Ye, L. , X. Xu , W. Qi , F. Chen , and G. Xia . 2024. “Risk Factors for Cancer‐Related Cognitive Impairment Among Individuals With Lung Cancer: A Systematic Review and Meta‐Analysis.” Supportive Care in Cancer 32, no. 10: 663. 10.1007/s00520-024-08873-w. 39287692

[brb370594-bib-0047] Zhang, W. , W. L. Wang , J. F. Hong , and Y. Chen . 2012. “A Study on the Critical Value of Hospital Anxiety and Depression Scale in Screening for Anxiety and Depression in Hospitalised Cancer Patients.” Journal of Nursing 19, no. 19: 1–4. 10.16460/j.issn1008-9969.2012.19.006.

[brb370594-bib-0048] Zhou, X. X. 2015. A Preliminary Study on the Reliability and Validity of the Chinese Version of the Simple Intelligent Mental State Examination Scale in Stroke Patients. Fujian University of Chinese Medicine.

[brb370594-bib-0049] Zhou, Z. , J. Ren , Q. Liu , et al. 2024. “A Nomogram for Predicting the Risk of Cancer‐Related Cognitive Impairment in Breast Cancer Patients Based on a Scientific Symptom Model.” Scientific Reports 14, no. 1: 14566. 10.1038/s41598-024-65406-5. 38914627 PMC11196746

